# Artificial Legs

**Published:** 1864-04

**Authors:** 


### ARTIFICIAL LEGS.

It will be observed by reference to advertisement page that Dr. De Forrest Douglass of Springfield, Mass., has invented and patented an artificial leg, which he offers to those who require, as possessing superior merits.—We have always looked upon the "Palmer Leg" as the only leg which could receive unqualified approval, and even that we found after a little longer acquaintance require too much repair; it was too often out of order, and cost too much to mend. When new it certainly works very finely, and the great objection to be urged against it was its liability to break down and require repair. We have carefully examined and have observed the working of one of the legs invented by Dr. Douglass, and we believe that it possesses all the perfections of an artificial leg, and is made in such manner as to retain them much longer than any other. It is claimed, and we believe rightfully, that they are more perfect and natural in their motions and more durable in construction than those of any other manufacturer. They are light, easily worn, beautifully natural in appearance, and every way attractive for those who require artificial legs. A specimen has been left in our office, which has been examined by a great many surgeons and receives unexceptional approval. Those physicians whose patients are requiring such instrument, are invited to call attention to this, for we believe that it certainly possesses advantages over all others. We have a little patient recently supplied with it, and it is truly matter of gratitude that human invention has been able to so nearly equal nature in the construction of legs.

